# Combination of taking neuropsychiatric medications and psychological distress in pregnant women, with behavioral problems in children at 2 years of age: The Tohoku Medical Megabank Project Birth and Three‐Generation Cohort Study

**DOI:** 10.1002/pcn5.226

**Published:** 2024-07-25

**Authors:** Ippei Takahashi, Taku Obara, Saya Kikuchi, Natsuko Kobayashi, Ryo Obara, Aoi Noda, Minoru Ohsawa, Tomofumi Ishikawa, Nariyasu Mano, Hidekazu Nishigori, Fumihiko Ueno, Genki Shinoda, Keiko Murakami, Masatsugu Orui, Mami Ishikuro, Hiroaki Tomita, Shinichi Kuriyama

**Affiliations:** ^1^ Division of Molecular Epidemiology, Graduate School of Medicine Tohoku University Sendai Japan; ^2^ Department of Preventive Medicine and Epidemiology, Tohoku Medical Megabank Organization Tohoku University Sendai Japan; ^3^ Department of Pharmaceutical Sciences Tohoku University Hospital Sendai Japan; ^4^ Department of Psychiatry Tohoku Graduate School of Medicine Sendai Japan; ^5^ Department of Psychiatry Tohoku University Hospital Sendai Japan; ^6^ Department of Psychiatry Kawasaki Kokoro Hospital Miyagi Japan; ^7^ Department of Education and Support for Regional Medicine Tohoku University Hospital Sendai Japan; ^8^ Department of Kampo Medicine Tohoku University Hospital Sendai Japan; ^9^ Laboratory of Clinical Pharmacy Tohoku University Graduate School of Pharmaceutical Sciences Sendai Japan; ^10^ Department of Development and Environmental Medicine Fukushima Medical University Graduate School of Medicine Fukushima Japan; ^11^ International Research, Institute of Disaster Science Tohoku University Sendai Japan

**Keywords:** behavioral problems, birth cohort, epidemiology, neuropsychiatric medications, psychological distress

## Abstract

**Aim:**

To examine the association of the combination of taking neuropsychiatric medications from the onset of pregnancy to mid‐pregnancy and maternal psychological distress at mid‐pregnancy, with children's behavioral problems.

**Methods:**

Neuropsychiatric medication use from the onset of pregnancy to mid‐pregnancy was defined by the self‐reported name of the neuropsychiatric medication in the questionnaire in early and mid‐pregnancy. Maternal psychological distress was defined by the Kessler Psychological Distress Scale (K6) ≥13 on the questionnaire in mid‐pregnancy. We classified the participants into four categories based on the combination of taking neuropsychiatric medications and psychological distress: “None,” “Medications only,” “K6 ≥ 13 only,” and “Both.” Children's behavioral problems were assessed using the Child Behavior Checklist for Ages 1½–5 (CBCL) at 2 years of age. The clinical ranges of the internalizing and externalizing scales of the CBCL were defined as behavioral problems. We conducted a multivariable logistic regression analysis to examine the associations between the four categories of maternal exposure and children's behavioral problems.

**Results:**

Compared with the “None” category (*n* = 9873), the “K6 ≥ 13 only” category (*n* = 308) was statistically significantly associated with internalizing and externalizing problems. In contrast, the “Medications only” (*n* = 93) and “Both” (*n* = 22) categories were not statistically significantly associated with internalizing and externalizing problems, although the point estimates of the odds ratio in the “Both” category were relatively high (1.58 for the internalizing problem and 2.50 for the externalizing problem).

**Conclusion:**

The category of mothers taking neuropsychiatric medications and having no psychological distress during pregnancy was not associated with children's behavioral problems in the present population.

## INTRODUCTION

Maternal psychiatric disorders commonly occur during pregnancy.^1^, ^2^, ^3^ The prevalence of depression during pregnancy in the global population has been reported to be 7.4% in early pregnancy, 12.8% in mid‐pregnancy, and 12.0% in late pregnancy.^1^ In addition, several studies have reported that maternal psychiatric symptoms, such as depression, anxiety, and psychological distress during pregnancy, are associated with behavioral problems in children,^4^, ^5^, ^6^, ^7^, ^8^, ^9^, ^10^, ^11^, ^12^, ^13^, ^14^, ^15^, ^16^ therefore maternal psychiatric symptoms are a serious problem that adversely affect both mothers and children.

For the treatment of psychiatric symptoms during pregnancy, pharmacotherapy is recommended after weighing up its benefits and risks.^17^ Strategies for psychopharmacological treatments are selected based on shared decision‐making between physicians and patients that considers the benefits (i.e., maintaining good mental health conditions) and risks (e.g., potential adverse effects on child neurological development^4^, ^5^, ^6^, ^7^, ^8^, ^9^, ^10^, ^11^, ^12^, ^13^, ^14^, ^15^, ^16^) from taking the medication.^17^ Psychosocial therapy is recommended for mild cases and antidepressant medications are recommended for moderate or severe cases.^17^, ^18^, ^19^ Although the impact of untreated depression on child outcomes is clear,^20^ the risks of using neuropsychiatric medications during pregnancy have been discussed and are inconclusive.^21^, ^22^, ^23^, ^24^, ^25^, ^26^, ^27^, ^28^, ^29^, ^30^, ^31^, ^32^, ^33^, ^34^ In addition, it has been suggested that the effects of medication on the fetus during pregnancy may not be due to the medication itself but rather to the mother's psychiatric disease and potential confounding factors.^35^, ^36^ However, a study showed that about half of Japanese pregnant women who were taking antidepressants discontinued them during pregnancy, therefore the possibility cannot be ruled out that people are actually focusing more on the risks than on the benefits of taking neuropsychiatric medications during pregnancy.^37^

Although the risks and benefits of treatment with neuropsychiatric medications during pregnancy have been discussed, to our knowledge only one study has considered the risks and benefits of neuropsychiatric medication use during pregnancy on a child's developmental outcome.^34^ A previous study examined the association of the combination of anxiety and whether or not neuropsychiatric medications were taken during pregnancy with infant auditory sensory gating, showing that in cases of anxiety during pregnancy there is an association between taking antidepressant medication and improved infant auditory sensory gating.^34^ The above study implies the potential benefit of antidepressant medication for children's development in utero with maternal anxiety. To appropriately weigh the risks and benefits of neuropsychiatric medications during pregnancy in clinical practice, it would be beneficial to accumulate evidence from birth cohort studies.

We aimed to examine the association of the combination of taking neuropsychiatric medications and maternal psychological distress (including some of the symptoms of depression and anxiety) during pregnancy with children's behavioral problems, which have been widely reported to be associated with psychiatric symptoms^4^, ^5^, ^6^, ^7^, ^8^, ^9^, ^10^, ^11^, ^12^, ^13^, ^14^, ^15^, ^16^ and taking neuropsychiatric medications^23^, ^26^ during pregnancy, in the Tohoku Medical Megabank Project Birth and Three‐Generation (TMM BirThree) Cohort Study.

## METHODS

### Study design and population

**Figure 1:**
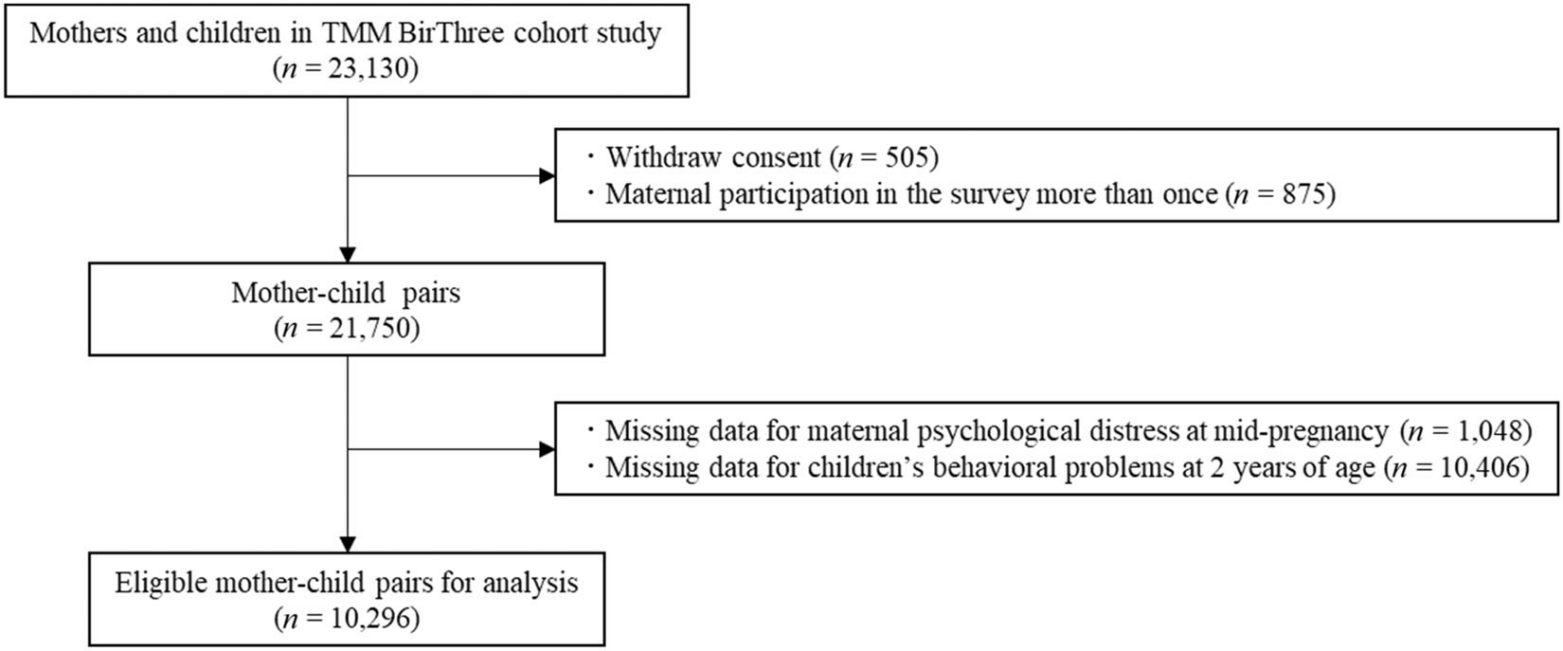
Participant exclusion criteria in this study.

This study was conducted using data from the TMM BirThree Cohort Study. Details of the TMM BirThree Cohort Study have been previously described.^38^, ^39^, ^40^, ^41^ Briefly, this study recruited pregnant women from approximately 50 obstetric clinics and hospitals in Miyagi and Iwate Prefectures, Japan, between July 2013 and March 2017.^39^ The details of the study were explained to potential participants by trained genome medical research coordinators and signed consent was obtained.^39^ The protocols of the TMM BirThree Cohort Study and the present study were reviewed and approved by the Institutional Review Board of the Tohoku Medical Megabank Organization (May 27, 2013, Approval No. 2013‐1‐103‐1 and December 21, 2020, Approval No. 2020‐4‐120). The exclusion criteria applied to 23,130 mother and child pairs were as follows: withdrawn informed consent (*n* = 505), multiple participation in the TMM BirThree Cohort Study (*n* = 875), missing data for maternal psychological distress in mid‐pregnancy (*n* = 1048), and missing information on children's behavioral problems at 2 years of age (*n* = 10,406). In total, 10,296 eligible mother–child pairs were included in the analysis (Figure 1). The present study followed the Strengthening the Reporting of Observational Studies in Epidemiology (STROBE) guidelines and the Declaration of Helsinki.

### Neuropsychiatric medications

Neuropsychiatric medications were assessed using a self‐report questionnaire during early and mid‐pregnancy. A questionnaire for early pregnancy was used to obtain information on medication use from the onset of pregnancy to early pregnancy, and a questionnaire for mid‐pregnancy was used to obtain information on medication use from early to mid‐pregnancy. Classification of neuropsychiatric medications used for neuropsychiatric symptoms was based on the Anatomical Therapeutic Chemical (ATC) classification system. The ATC classification codes for the neuropsychiatric medications used in the present study were as follows: N03A (antiepileptics), NO5A (antipsychotics), NO5B (anxiolytics), NO5C (hypnotics and sedatives), NO6A (antidepressants), and NO6B (psychostimulants, agents used for attention deficit hyperactivity disorder, and nootropics). In addition, neuropsychiatric medications and Japanese Kampo medicines used for neuropsychiatric symptoms, but not covered by the ATC classification system, were also selected: flutoprazepam, gamma‐oryzanol, rilmazafone hydrochloride hydrate, hangekobokuto, ryokeijutsukanto, kososan, kambakutaisoto, keishikaryukotsuboreito, nyoshinsan, saikokeishikankyoto, yokukansan, and yokukansankachimpihange. Pregnant women who self‐reported taking these medications in the early‐ or mid‐pregnancy questionnaires were defined as taking neuropsychiatric medications in the present study. The definitions of neuropsychiatric medications for neuropsychiatric symptoms were selected by psychiatrists (S.K., N.K., and M. Ohsawa) and pharmacists (T.O., R.O., and A.N.).

### Maternal psychological distress

To assess maternal psychological distress during mid‐pregnancy, we used the Japanese version of the Kessler Psychological Distress Scale (K6).^42^, ^43^, ^44^, ^45^, ^46^ The K6 is a short screening scale comprising six questions. Kessler et al. developed the K6,^42^ and Furukawa et al. developed a Japanese version of the K6.^43^ We defined psychological distress as a K6 score of >13 points. This cutoff score has been proposed to operationalize the definition of serious mental illness, which is defined as meeting the diagnostic criteria for a DSM‐IV disorder in the past 12 months and experiencing significant impairment.^44^, ^45^ Furthermore, we classified the mothers into four categories to compare the effects of each category's combination of neuropsychiatric medication use during pregnancy and psychological distress on child behavioral problems:

(1) Did not take neuropsychiatric medications from the onset of pregnancy to mid‐pregnancy and did not have psychological distress at mid‐pregnancy (None); (2) took neuropsychiatric medications from onset of pregnancy to mid‐pregnancy and did not have psychological distress at mid‐pregnancy (Medications only); (3) did not take neuropsychiatric medications from onset of pregnancy to mid‐pregnancy and had psychological distress at mid‐pregnancy (K6 ≥ 13 only); and (4) took neuropsychiatric medications from onset of pregnancy to mid‐pregnancy and had psychological distress at mid‐pregnancy (Both).

### Children's behavioral problems

To assess behavioral problems in children aged 2 years, we used the Child Behavior Checklist for Ages 1½–5 (CBCL), which was responded to by the caregiver.^47^, ^48^ The CBCL consists of 100 items divided into seven syndrome scales (emotionally reactive, anxious/depressed, somatic complaints, withdrawn, sleep problems, attention problems, and aggressive behavior).^47^, ^48^ In addition, the scale for internalizing problems was defined as the total score for emotionally reactive, anxious/depressed, somatic complaints, and withdrawn problems. The scale for externalizing problems was defined as the total score for attention problems and aggressive behavior.^47^, ^48^ The *T* score (mean 50, standard deviation 10) for each scale was calculated and standardized for Japanese children, and a standardized *T* score of 64 or higher indicated the clinical range.^48^ This study defined the clinical range of internalizing and externalizing problems (standardized *T* score ≥64) as having behavioral problems.

### Covariates

We selected information that may influence the association between neuropsychiatric medication use and maternal psychological distress during pregnancy and children's behavioral problems, referring to previous studies.^4^, ^5^, ^6^, ^7^, ^8^, ^9^, ^10^, ^11^, ^12^, ^13^, ^14^, ^15^, ^16^, ^21^, ^22^, ^23^, ^24^, ^25^, ^26^, ^27^, ^28^, ^29^, ^30^, ^31^, ^32^, ^33^, ^34^ Information on children's sex was obtained from their birth records. Information on maternal age at delivery and parity (never, once, or more) was obtained from medical records. Maternal age was divided into four categories (<25, 25–29, 30–34, and ≥35 years). Information on maternal alcohol consumption (never, former, current), maternal cigarette smoking (never, stopped before pregnancy, stopped after pregnancy, current), and paternal cigarette smoking (never, stopped before pregnancy, stopped after pregnancy, current) was collected from the questionnaire during early pregnancy. Information on household income (<4,000,000, 4,000,000–5,999,999, ≥6,000,000 Japanese yen/year) was gathered from the mid‐pregnancy questionnaire. Maternal educational attainment data (high school graduate or less, junior college or vocational college graduate, university graduate or above, and others) were gathered from a questionnaire response after 1 year postpartum. Postpartum psychological distress is also an important factor affecting child development. However, the purpose of this study was to infer the causality of the combination of neuropsychiatric medication and psychological stress during pregnancy and children's behavioral problems. In this context, postpartum psychological distress is positioned as a intermediate factor in the above causal inference, thus we did not adjust for postpartum psychological distress as a covariate.

### Statistical analysis

We described the characteristics of the participants according to the four categories of taking neuropsychiatric medications and experiencing psychological distress during pregnancy. Characteristics are presented as frequencies and percentages for categorical variables and median and interquartile range (IQR) for numerical variables. In addition, because only about 47.3% (*n* = 10,296) of the mother–child pairs who agreed to participate in the TMM BirThree cohort study (*n* = 21,750) were included in our analysis, we performed a dropout analysis, a comparison of the characteristics of the participants in the analysis with those of the excluded participants to examine the possibility of selection bias. The characteristics of the participants according to the two groups of participants included (*n* = 10,296) and participants not included (*n* = 11,454) were described, and P‐values were obtained from *χ* ^2^ tests comparing participant characteristics. The variables used in the dropout analysis were as follows: maternal age at delivery, parity, maternal alcohol drink, maternal smoking, maternal educational attainment, household income, paternal smoking, and children's sex. As the main analysis, we conducted a multivariable logistic regression analysis examining the association of four categories of taking neuropsychiatric medications and psychological distress during pregnancy with the children's behavioral problems at 2 years of age to estimate odds ratios (ORs) and 95% confidence intervals (CIs). Missing covariates were imputed through multiple imputations by chained equations using exposure, outcome, and covariates in each model.^49^ Twenty sets of quasicomplete data were independently analyzed in multivariable analyses and the estimates were integrated.^49^ We also performed a sensitivity analysis in which the cutoffs for K6 were changed to an even more moderate K6 ≥ 5 and 9. All statistical analyses were performed using R (v.4.0.2) and 95% CIs not crossing 1.00 were considered statistically significant.

## RESULTS

### Participant characteristics

Participant characteristics according to the four categories of neuropsychiatric medications and maternal psychological distress are shown in Table 1. Among the 10,296 mothers, 9,873 (95.9%) were assessed as “None,” 93 (0.9%) as “Medications only,” 308 (3.0%) as “K6 ≥ 13 only,” and 22 (0.2%) as “Both.” Among the 10,296 children, 1158 (11.2%) were assessed with externalizing problems and 847 (8.2%) were assessed with internalizing problems. Regarding taking neuropsychiatric medications, in total 115 (“Medications only” + “Both”) pregnant women had taken neuropsychiatric medications from the onset of pregnancy to mid‐pregnancy. The continuous K6 scores were higher for “Both,” “K6 ≥ 13 only,” “Medications only,” and “None,” in that order. A detailed summary of the neuropsychiatric medications taken by the 115 pregnant women is given in Supporting Information Table S1. A detailed summary of the number of mothers who took neuropsychiatric medications during pregnancy for psychological distress during mid‐pregnancy is given in Supporting Information Table S2.

**Table 1:** Characteristics of participants according to the four categories of taking neuropsychiatric medications and psychological distress in pregnancy.

	Total	None	Medications only	K6 ≥ 13 only	Both
	*n* = 10,296	*n* = 9873	*n* = 93	*n* = 308	*n* = 22
*K6 score*					
Median (IQR)	2.00 (0.05, 5.00)	2.00 (0.00, 5.00)	4.00 (2.00, 7.00)	15.00 (14.00, 17.00)	17.00 (14.25, 19.75)
*Maternal age, n (%)*					
<25 (years)	548 (5.3)	508 (5.1)	3 (3.2)	34 (11.0)	3 (13.6)
25–30 (years)	2475 (24.0)	2335 (23.7)	27 (29.0)	107 (34.7)	6 (27.3)
30–35 (years)	3946 (38.3)	3815 (38.6)	29 (31.2)	96 (31.2)	6 (27.3)
>35 (years)	3327 (32.3)	3215 (32.6)	34 (36.6)	71 (23.1)	7 (31.8)
*Parity, n* (%)					
Nullpara	4876 (47.4)	4645 (47.0)	55 (59.1)	166 (53.9)	10 (45.5)
Multipara	5397 (52.4)	5205 (52.7)	38 (40.9)	142 (46.1)	12 (54.5)
*Maternal alcohol drink, n (%)*					
Never	4655 (45.2)	4459 (45.2)	46 (49.5)	142 (46.1)	8 (36.4)
Fomer	3472 (33.7)	3321 (33.6)	31 (33.3)	110 (35.7)	10 (45.5)
Current	2119 (20.6)	2044 (20.7)	16 (17.2)	55 (17.9)	4 (18.2)
*Maternal smoking, n (%)*					
Never	6554 (63.7)	6329 (64.1)	58 (62.4)	160 (51.9)	7 (31.8)
Stopped before pregnancy	2415 (23.5)	2299 (23.3)	19 (20.4)	89 (28.9)	8 (36.4)
Stopped after pregnancy	1108 (10.8)	1044 (10.6)	12 (12.9)	47 (15.3)	5 (22.7)
Current	162 (1.6)	145 (1.5)	4 (4.3)	11 (3.6)	2 (9.1)
*Educational attainment, n (%)*					
High school graduate or less	2820 (27.4)	2659 (26.9)	29 (31.2)	124 (40.3)	8 (36.4)
Junior college or vocational college graduate	3570 (34.7)	3440 (34.8)	28 (30.1)	96 (31.2)	6 (27.3)
University graduate or above	2802 (27.2)	2712 (27.5)	26 (28.0)	61 (19.8)	3 (13.6)
Others	24 (0.2)	23 (0.2)	1 (1.1)	0 (0.0)	0 (0.0)
*Income, n (%)*					
<4,000,000 (JPY/year)	3322 (32.3)	3122 (31.6)	36 (38.7)	153 (49.7)	11 (50.0)
4,000,000 to <5,999,999 (JPY/year)	3325 (32.3)	3222 (32.6)	24 (25.8)	73 (23.7)	6 (27.3)
≥6,000,000 (JPY/year)	3238 (31.4)	3144 (31.8)	27 (29.0)	64 (20.8)	3 (13.6)
*Paternal smoking, n (%)*					
Never	3102 (30.1)	2989 (30.3)	22 (23.7)	86 (27.9)	5 (22.7)
Stopped before pregnancy	2490 (24.2)	2401 (24.3)	21 (22.6)	66 (21.4)	2 (9.1)
Stopped after pregnancy	274 (2.7)	261 (2.6)	6 (6.5)	6 (1.9)	1 (4.5)
Current	4327 (42.0)	4125 (41.8)	43 (46.2)	145 (47.1)	14 (63.6)
*Child's gender, n (%)*					
Male	5306 (51.5)	5079 (51.4)	48 (51.6)	168 (54.5)	11 (50.0)
*Behavioral problems, n (%)*					
Externalizing problems	1158 (11.2)	1059 (10.7)	12 (12.9)	81 (26.3)	6 (27.3)
Internalizing problems	847 (8.2)	778 (7.9)	10 (10.8)	56 (18.2)	3 (13.6)

K6, Kessler Psychological Distress Scale; IQR, interquartile range; JPY, Japanese yen.

### Comparison of characteristics of included and not included participants

The results of the dropout analysis comparing the characteristics of included and not‐included participants are shown in Supporting Information Table S3. Mothers excluded from the analysis were younger, less educated, and had lower incomes, lower rates of current alcohol drinking, higher rates of current smoking, and higher rates of partner's current smoking.

### Multivariable logistic regression analysis

The associations of the four categories of neuropsychiatric medication use and maternal psychological distress with children's behavioral problems at 2 years of age investigated by multivariable logistic regression are shown in Table 2. After adjusting for possible confounders, associations are shown for the “K6 ≥ 13 only” category with children's externalizing problems (OR = 2.54, 95% CI 1.94–3.32 for K6 ≥ 13 only vs. None) and internalizing problems (OR = 2.30, 95% CI 1.69–3.12 for K6 ≥ 13 only vs. None) at 2 years of age but are not shown for women in the “Medications only” and “Both” groups. The sensitivity analysis results using cutoff scores of 5 and 9 for the K6 score are given in Supporting Information Tables S4 and S5, respectively. From the results of sensitivity analysis, we confirmed the association in the categories of “K6 ≥ 5 only” and “Both” with externalizing and internalizing problems in Supporting Information Table S4. In addition, the “K6 ≥ 9 only” category was associated with children's externalizing and internalizing problems as shown in Supporting Information Table S5. When we excluded children diagnosed with autism spectrum disorder, cerebral palsy, or chromosomal abnormalities by age 2 (*n* = 6), the results did not change.

**Table 2:** The association of the four categories of taking neuropsychiatric medications and maternal psychological distress (K6 ≥ 13) during pregnancy with children's behavioral problems at 2 years of age by multivariate logistic regression.

Children's behavioral problems at aged 2 years	None (*n* = 9873)		Medications only (*n* = 93)		K6 ≥ 13 only (*n* = 308)		Both (*n* = 22)	
	Crude OR (95% CI)	Adjusted OR (95% CI)^a^	Crude OR (95% CI)	Adjusted OR (95% CI)^a^	Crude OR (95% CI)	Adjusted OR (95% CI)^a^	Crude OR (95% CI)	Adjusted OR (95% CI)^a^
Externalizing problems	Ref		1.23 (0.64–2.18)	1.14 (0.62–2.12)	2.97 (2.27–3.84)	2.54 (1.94–3.32)	3.12 (1.12–7.61)	2.50 (0.96–6.54)
Internalizing problems	Ref		1.41 (0.68–2.59)	1.22 (0.63–2.39)	2.60 (1.91–3.47)	2.30 (1.69–3.12)	1.85 (0.43–5.43)	1.58 (0.45–5.50)

K6, Kessler Psychological Distress Scale; 95% CI, 95% confidence interval; OR, odds ratio.

^a^ Adjusted for maternal age at delivery, parity, educational attainment, household income, maternal alcohol intake, maternal cigarette smoking, paternal cigarette smoking, and child's sex.

## DISCUSSION

The present study examined whether there was an association between mothers who had taken neuropsychiatric medications from the onset of their pregnancy to mid‐pregnancy (with or without maternal psychological distress defined as K6 ≥ 13 at mid‐pregnancy) and their children's behavioral problems at age 2 years. The definitions of the use of neuropsychiatric medication as “None,” “Medications only,” “K6 ≥ 13 only,” and “Both” are explained above. As a result, the category of “K6 ≥ 13 only” was associated with both internalizing and externalizing problems, with the “None” category as the reference, while the categories of “Medications only” and "Both" were not associated with internalizing and externalizing problems.

The association found for the category of “K6 ≥ 13 only” is consistent with the findings of previous studies examining the association between maternal psychiatric symptoms during pregnancy and behavioral problems in children.^4^, ^5^, ^6^, ^7^, ^8^, ^9^, ^10^, ^11^, ^12^, ^13^, ^14^, ^15^, ^16^ This study also confirmed this association by a sensitivity analysis using K6 cutoff scores of 5 and 9. While there are several hypothesized mechanisms by which maternal psychiatric symptoms during pregnancy affect the development of the child,^50^ the major consideration is through the downregulation of 11β‐hydroxysteroid dehydrogenase type 2 (11β‐HSD2) gene expression in the placenta.^51^ Normally, placental 11β‐HSD2 protects the fetus from cortisol exposure by inactivating cortisol. However, 11β‐HSD2 gene expression has been reported to be downregulated in the placenta by maternal anxiety.^51^ Exposure of the fetus to high cortisol levels in utero may cause reprogramming of the fetal hypothalamus (pituitary) axis, which may adversely affect child development.^52^ The present study reaffirms the importance of screening for psychological distress during pregnancy and highlights the importance of adequate management of psychiatric distress using neuropsychiatric medications.

No association was found in the “Medications only” category in the present study. One possible explanation for this is that neuropsychiatric medications themselves might not be significantly associated with children's behavioral problems in the present population. However, the present findings cannot be taken as an argument that maternal neuropsychiatric medications during pregnancy benefit children's behavioral problems because the use of neuropsychiatric medications has a wide range of indications and situations, and is not always directly related to a reduction in psychological distress. The results of this study suggest the importance of accumulating evidence on the risks and benefits of taking neuropsychiatric medications during pregnancy, considering maternal mental health conditions.

Even though the “Both” category has the highest K6 scores, no association was found between this category and children's behavioral problems. One of the primary explanations for this is the small sample size of the “Both” category. In addition, point estimates of the odds ratio for the “Both” category are higher (OR = 2.50 for the externalizing problems and 1.58 for the internalizing problems). Similarly, a sensitivity analysis with a cutoff of 9 points for K6 confirms no association, but the point estimate is higher (OR = 1.72 for the internalizing problems and 1.76 for the externalizing problems), and a sensitivity analysis with a cutoff of 5 points with the largest sample size in category “Both” confirms an association with externalizing and internalizing problems. From the above, it is likely that an association would be detected if the sample size for the “Both” category increased. In such cases, maternal psychological distress might not be controlled by neuropsychological medications, therefore it may be necessary to review the treatment of severe maternal psychiatric symptoms and the environment surrounding pregnant women. Although the sample size for both categories was small, birth cohorts that prospectively collected maternal psychiatric symptoms and medications during pregnancy were limited. The TMM BirThree cohort study with 10,296 participants was one of the best cohorts to conduct this study.

To the best of our knowledge, this is the first study to examine the association of a combination of neuropsychiatric medications and maternal psychological distress during pregnancy with behavioral problems in children. The strength of this study is the use of longitudinal birth cohort data containing a rich set of variables, which allowed us to examine the association while prospectively adjusting for several confounding factors. However, this study had several limitations. First, because we focused on psychological distress as an exposure factor, our study is limited in its comparability with previous studies that have examined the association between maternal depression or anxiety during pregnancy and behavioral problems in children. Additionally, psychological distress reflects aspects of both psychiatric disorders targeted by neuropsychiatric medications and a wide range of psychological conditions that are not targeted by neuropsychiatric medication. Second, we could not focus on specific neuropsychiatric medications such as selective serotonin reuptake inhibitors. This was due to the small number of pregnant women taking neuropsychiatric medications in the present population. Future studies with larger sample sizes are required to overcome this limitation. Third, of the mother–child pairs who agreed to participate in the TMM BirThree cohort study, only about 47.3% were included in our analysis, affecting our results' external validity. In fact, the dropout analysis confirmed differences in the characteristics of included and excluded participants (Supporting Information Table S3). The characteristics of the excluded mother–child pairs were similar to those of the mothers with psychological distress (Table 1 and Supporting Information Table S3). Under these circumstances, the results of this study may underestimate the association between maternal psychological distress and child behavioral problems. Future studies analyzing populations with varying characteristics will provide a more detailed architecture of the association between maternal psychological distress and behavioral problems. Fourth, we used data on self‐reported neuropsychiatric medication intake, therefore we cannot rule out the possibility that the mothers were prescribed but not taking neuropsychiatric medications. Fifth, no information was collected on psychological support for mothers other than medication, such as psychosocial therapy. It cannot be ruled out that psychological support other than medication would adjust the estimation in this study. Finally, the CBCL was filled out by the caregiver, therefore when a mother with psychological distress responds to the CBCL, the possibility of systematic errors in the responses cannot be ruled out.

## CONCLUSION

Compared to the group of pregnant women without neuropsychiatric medications and psychological distress, the group of pregnant women with psychological distress but without neuropsychiatric medication had a higher risk of behavioral problems in their children at 2 years of age. In contrast, the group of pregnant women taking neuropsychiatric medications but without psychological distress might have no increased risk of children's behavioral problems at 2 years of age.

## AUTHOR CONTRIBUTIONS

Ippei Takahashi, Taku Obara, Saya Kikuchi, Natsuko Kobayashi, and Hiroaki Tomita were responsible for the study conception, design, and interpretation of the results. Ippei Takahashi was responsible for the analysis and drafting of the manuscript. Taku Obara, Aoi Noda, Fumihiko Ueno, Genki Shinoda, Keiko Murakami, Masatsugu Orui, Mami Ishikuro, and Shinichi Kuriyama contributed to data collection. Taku Obara provided advice on essential intellectual content and helped draft the manuscript. All the authors have read and approved the final version of the manuscript.

## CONFLICT OF INTEREST STATEMENT

Minoru Ohsawa belongs to the Department of Kampo and Integrative Medicine at Tohoku University School of Medicine. The department received a grant from Tsumura & Co., a Japanese manufacturer of Kampo Medicine. The grant was used according to the rules of Tohoku University. Potential conflicts of interest were addressed and managed appropriately by the Tohoku University Benefit Reciprocity Committee. Additionally, Tomofumi Ishikawa is an employee of Pfizer R&D in Japan and a research collaborator at Tohoku University, Japan. They contributed to the present study independent of Pfizer R&D Japan.

## ETHICS APPROVAL STATEMENT

The TMM BirThree Cohort Study protocol and the present study were performed in line with the principles of the Declaration of Helsinki and approval was granted by the Ethics Committee of Tohoku University Tohoku Medical Megabank Organization (May 27, 2013, Approval No. 2013‐1‐103‐1 and December 21, 2020, Approval No. 2020‐4‐120).

## PATIENT CONSENT STATEMENT

The details of the TMM BirThree Cohort Study were explained to potential participants by trained genomic medical research coordinators and signed consent was obtained.

## CLINICAL TRIAL REGISTRATION

This study is an observational study, not a clinical study.

## Supporting information

Supporting information.

## Data Availability

The data supporting the findings of this study are available from the TMM Biobank. Restrictions apply to the availability of these data, which were used under license for the current study and hence are not publicly available. Data are available from the authors upon reasonable request and with permission from the TMM Biobank.

## References

[1] Bennett HA , Einarson A , Taddio A , Koren G , Einarson TR . Prevalence of depression during pregnancy: systematic review. Obstet Gynecol. 2004;103:698–709. 15051562 10.1097/01.AOG.0000116689.75396.5f

[2] Tokumitsu K , Sugawara N , Maruo K , Suzuki T , Shimoda K , Yasui‐Furukori N . Prevalence of perinatal depression among Japanese women: a meta‐analysis. Ann Gen Psychiatry. 2020;19:41. 32607122 10.1186/s12991-020-00290-7 PMC7320559

[3] Kikuchi S , Murakami K , Obara T , Ishikuro M , Ueno F , Noda A , et al. One‐year trajectories of postpartum depressive symptoms and associated psychosocial factors: Findings from the Tohoku Medical Megabank Project Birth and Three‐Generation Cohort Study. J Affect Disord. 2021;295:632–638. 34509778 10.1016/j.jad.2021.08.118

[4] Gressier F , Letranchant A , Glatigny‐Dallay E , Falissard B , Sutter‐Dallay AL . Negative impact of maternal antenatal depressive symptoms on neonate's behavioral characteristics. Eur Child Adolesc Psychiatry. 2020;29:515–526. 31297657 10.1007/s00787-019-01367-9

[5] Faleschini S , Rifas‐Shiman SL , Tiemeier H , Oken E , Hivert MF . Associations of prenatal and postnatal maternal depressive symptoms with offspring cognition and behavior in mid‐childhood: a prospective cohort study. Int J Environ Res Public Health. 2019;16:1007. 30897718 10.3390/ijerph16061007 PMC6466510

[6] Gjerde LC , Eilertsen EM , Reichborn‐Kjennerud T , McAdams TA , Zachrisson HD , Zambrana IM , et al. Maternal perinatal and concurrent depressive symptoms and child behavior problems: a sibling comparison study. J Child Psychol Psychiatry. 2017;58:779–786. 28229455 10.1111/jcpp.12704 PMC5484352

[7] Woolhouse H , Gartland D , Mensah F , Giallo R , Brown S . Maternal depression from pregnancy to 4 years postpartum and emotional/behavioural difficulties in children: results from a prospective pregnancy cohort study. Arch Women's Ment Health. 2016;19:141–151. 26271281 10.1007/s00737-015-0562-8

[8] Giallo R , Woolhouse H , Gartland D , Hiscock H , Brown S . The emotional–behavioural functioning of children exposed to maternal depressive symptoms across pregnancy and early childhood: a prospective Australian pregnancy cohort study. Eur Child Adolesc Psychiatry. 2015;24:1233–1244. 25572869 10.1007/s00787-014-0672-2

[9] O'Donnell KJ , Glover V , Barker ED , O'Connor TG . The persisting effect of maternal mood in pregnancy on childhood psychopathology. Dev Psychopathol. 2014;26:393–403. 24621564 10.1017/S0954579414000029

[10] Park S , Kim BN , Kim JW , Shin MS , Yoo HJ , Lee J , et al. Associations between maternal stress during pregnancy and offspring internalizing and externalizing problems in childhood. Int J Ment Health Syst. 2014;8:44. 25926872 10.1186/1752-4458-8-44 PMC4414378

[11] Giles LC , Davies MJ , Whitrow MJ , Warin MJ , Moore V . Maternal depressive symptoms and childcare during toddlerhood relate to child behavior at age 5 years. Pediatrics. 2011;128:e78–e84. 21669897 10.1542/peds.2010-3119

[12] Fihrer I , McMahon CA , Taylor AJ . The impact of postnatal and concurrent maternal depression on child behaviour during the early school years. J Affect Disord. 2009;119:116–123. 19342104 10.1016/j.jad.2009.03.001

[13] Yamada M , Tanaka K , Arakawa M , Miyake Y . Perinatal maternal depressive symptoms and risk of behavioral problems at five years. Pediatr Res. 2022;92(1):315–321. 34465880 10.1038/s41390-021-01719-9

[14] Kingston D , Kehler H , Austin MP , Mughal MK , Wajid A , Vermeyden L , et al. Trajectories of maternal depressive symptoms during pregnancy and the first 12 months postpartum and child externalizing and internalizing behavior at three years. PLoS One. 2018;13:e0195365. 29652937 10.1371/journal.pone.0195365 PMC5898728

[15] Takahashi I , Obara T , Kikuchi S , Kobayashi M , Ishikuro M , Murakami K , et al. Association between maternal psychological distress and children's neurodevelopment in offspring aged 4 years in Japan: the Tohoku Medical Megabank Project Birth and Three‐Generation Cohort Study. J Paediatr Child Health. 2023;59(3):548–554. 36751990 10.1111/jpc.16353

[16] Takahashi I , Murakami K , Kobayashi M , Kikuchi S , Igarashi A , Obara T , et al. Association of maternal psychological distress and the use of childcare facilities with children's behavioral problems: the Tohoku Medical Megabank Project Birth and Three‐Generation Cohort Study. BMC Psychiatry. 2022;22(1):693. 36357866 10.1186/s12888-022-04330-2 PMC9650864

[17] 2020 Evidence‐Based Guidelines for Midwifery Care Guidelines Committee of the Japan Academy of Midwifery. J Japan Acad Midwifery. 2020. Accessed June 8, 2024. https://www.jyosan.jp/uploads/files/journal/210311-JJAM_2020Evidence-Based_Duidelines_Midwifery_Care_Final2.pdf

[18] Kobayashi N , Nemoto H , Seto M , Sato S , Kikuchi S , Honda N , et al. Experiences of perinatal women and public healthcare providers in a community affected by the great east Japan earthquake and tsunami: concerns that must be considered for the mental healthcare of perinatal women in postdisaster settings. Int J Disaster Risk Reduct. 2020;51:101767.

[19] Seto M , Nemoto H , Kobayashi N , Kikuchi S , Honda N , Kim Y , et al. Post‐disaster mental health and psychosocial support in the areas affected by the Great East Japan Earthquake: a qualitative study. BMC Psychiatry. 2019;19:261. 31455275 10.1186/s12888-019-2243-z PMC6712862

[20] Jarde A , Morais M , Kingston D , Giallo R , MacQueen GM , Giglia L , et al. Neonatal outcomes in women with untreated antenatal depression compared with women without depression: a systematic review and meta‐analysis. JAMA Psychiatry. 2016;73(8):826–837. 27276520 10.1001/jamapsychiatry.2016.0934

[21] Corti S , Pileri P , Mazzocco MI , Mandò C , Moscatiello AF , Cattaneo D , et al. Neonatal outcomes in maternal depression in relation to intrauterine drug exposure. Front Pediatr. 2019;7:309. 31403037 10.3389/fped.2019.00309 PMC6676795

[22] Johnson KC , LaPrairie JL , Brennan PA , Stowe ZN , Newport DJ . Prenatal antipsychotic exposure and neuromotor performance during infancy. Arch Gen Psychiatry. 2012;69(8):787–794. 22474072 10.1001/archgenpsychiatry.2012.160 PMC4714592

[23] Brandlistuen RE , Ystrom E , Eberhard‐Gran M , Nulman I , Koren G , Nordeng H . Behavioural effects of fetal antidepressant exposure in a Norwegian cohort of discordant siblings. Int J Epidemiol. 2015;44(4):1397–1407. 25873178 10.1093/ije/dyv030 PMC4588862

[24] Clements CC , Castro VM , Blumenthal SR , Rosenfield HR , Murphy SN , Fava M , et al. Prenatal antidepressant exposure is associated with risk for attention‐deficit hyperactivity disorder but not autism spectrum disorder in a large health system. Mol Psychiatry. 2015;20(6):727–734. 25155880 10.1038/mp.2014.90 PMC4427538

[25] Hanley GE , Brain U , Oberlander TF . Infant developmental outcomes following prenatal exposure to antidepressants, and maternal depressed mood and positive affect. Early Hum Dev. 2013;89(8):519–524. 23384962 10.1016/j.earlhumdev.2012.12.012

[26] Oberlander TF , Papsdorf M , Brain UM , Misri S , Ross C , Grunau RE . Prenatal effects of selective serotonin reuptake inhibitor antidepressants, serotonin transporter promoter genotype (SLC6A4), and maternal mood on child behavior at 3 years of age. Arch Pediatr Adolesc Med. 2010;164(5):444–451. 20439795 10.1001/archpediatrics.2010.51

[27] Croen LA . Antidepressant use during pregnancy and childhood autism spectrum disorders. Arch Gen Psychiatry. 2011;68(11):1104–1112. 21727247 10.1001/archgenpsychiatry.2011.73

[28] Rai D , Lee BK , Dalman C , Golding J , Lewis G , Magnusson C . Parental depression, maternal antidepressant use during pregnancy, and risk of autism spectrum disorders: population based case‐control study. BMJ. 2013;346:f2059. 23604083 10.1136/bmj.f2059 PMC3630989

[29] Pedersen LH , Henriksen TB , Olsen J . Fetal exposure to antidepressants and normal milestone development at 6 and 19 months of age. Pediatrics. 2010;125(3):e600–e608. 20176667 10.1542/peds.2008-3655

[30] Casper RC , Fleisher BE , Lee‐Ancajas JC , Gilles A , Gaylor E , DeBattista A , et al. Follow‐up of children of depressed mothers exposed or not exposed to antidepressant drugs during pregnancy. J Pediatr. 2003;142(4):402–408. 12712058 10.1067/mpd.2003.139

[31] Figueroa R . Use of antidepressants during pregnancy and risk of attention‐deficit/hyperactivity disorder in the offspring. J Dev Behav Pediatr. 2010;31(8):641–648. 20613624 10.1097/DBP.0b013e3181e5ac93

[32] Casper RC , Gilles AA , Fleisher BE , Baran J , Enns G , Lazzeroni LC . Length of prenatal exposure to selective serotonin reuptake inhibitor (SSRI) antidepressants: effects on neonatal adaptation and psychomotor development. Psychopharmacology. 2011;217(2):211–219. 21499702 10.1007/s00213-011-2270-z

[33] Yamamoto‐Sasaki M , Yoshida S , Takeuchi M , Tanaka‐Mizuno S , Ogawa Y , Furukawa TA , et al. Association between antidepressant use during pregnancy and autism spectrum disorder in children: a retrospective cohort study based on Japanese claims data. Matern Health Neonatol Perinatol. 2019;5:1. 30652008 10.1186/s40748-018-0096-y PMC6327597

[34] Hunter SK , Mendoza JH , D'Anna K , Zerbe GO , McCarthy L , Hoffman C , et al. Antidepressants may mitigate the effects of prenatal maternal anxiety on infant auditory sensory gating. Am J Psychiatry. 2012;169(6):616–624. 22581104 10.1176/appi.ajp.2012.11091365 PMC3640273

[35] Mezzacappa A , Lasica PA , Gianfagna F , Cazas O , Hardy P , Falissard B , et al. Risk for autism spectrum disorders according to period of prenatal antidepressant exposure: a systematic review and meta‐analysis. JAMA Pediatrics. 2017;171(6):555–563. 28418571 10.1001/jamapediatrics.2017.0124

[36] Suarez EA , Bateman BT , Hernández‐Díaz S , Straub L , Wisner KL , Gray KJ , et al. Association of antidepressant use during pregnancy with risk of neurodevelopmental disorders in children. JAMA Intern Med. 2022;182:1149. 36190722 10.1001/jamainternmed.2022.4268 PMC9531086

[37] Ishikawa T , Obara T , Kikuchi S , Kobayashi N , Miyakoda K , Nishigori H , et al. Antidepressant prescriptions for prenatal and postpartum women in Japan: a health administrative database study. J Affect Disord. 2020;264:295–303. 10.1016/j.jad.2020.01.016 32056764

[38] Kuriyama S , Yaegashi N , Nagami F , Arai T , Kawaguchi Y , Osumi N , et al. The Tohoku medical Megabank project: design and mission. J Epidemiol. 2016;26(9):493–511. 27374138 10.2188/jea.JE20150268 PMC5008970

[39] Kuriyama S , Metoki H , Kikuya M , Obara T , Ishikuro M , Yamanaka C , et al. Cohort profile: Tohoku Medical Megabank Project Birth and Three‐Generation Cohort Study (TMM BirThree Cohort Study): rationale, progress and perspective. Int J Epidemiol. 2020;49(1):18–19m. 31504573 10.1093/ije/dyz169 PMC7124511

[40] Ishikuro M , Obara T , Osanai T , Yamanaka C , Sato Y , Mizuno S , et al. Strategic methods for recruiting grandparents: the Tohoku Medical Megabank Birth and Three‐Generation Cohort Study. Tohoku J Exp Med. 2018;246(2):97–105. 30333380 10.1620/tjem.246.97

[41] Sugawara J , Ishikuro M , Obara T , Onuma T , Murakami K , Kikuya M , et al. Maternal baseline characteristics and perinatal outcomes: the Tohoku Medical Megabank project birth and three‐generation cohort study. J Epidemiol. 2022;32(2):69–79. 33041318 10.2188/jea.JE20200338 PMC8761563

[42] Kessler RC , Andrews G , Colpe LJ , HIRIPI E , MROCZEK DK , NORMAND SLT , et al. Short screening scales to monitor population prevalences and trends in non‐specific psychological distress. Psychol Med. 2002;32:959–976. 12214795 10.1017/s0033291702006074

[43] Furukawa TA , Kawakami N , Saitoh M , Ono Y , Nakane Y , Nakamura Y , et al. The performance of the Japanese version of the K6 and K10 in the World Mental Health Survey Japan. Int J Methods Psychiatr Res. 2008;17:152–158. 18763695 10.1002/mpr.257 PMC6878390

[44] Prochaska JJ , Sung HY , Max W , Shi Y , Ong M . Validity study of the K6 scale as a measure of moderate mental distress based on mental health treatment need and utilization. Int J Methods Psychiatr Res. 2012;21(2):88–97. 10.1002/mpr.1349 22351472 PMC3370145

[45] Kessler RC , Berglund PA , Zhao S , Leaf PJ , Kouzjs AC , Bruce ML . The 12 month prevalence and correlates of serious mental illness. In: Mental health. Washington, DC: US Government Printing Office; 1996. p. 59–70.

[46] Sakurai K , Nishi A , Kondo K , Yanagida K , Kawakami N . Screening performance of K6/K10 and other screening instruments for mood and anxiety disorders in Japan. Psychiatry Clin Neurosci. 2011;65:434–441. 21851452 10.1111/j.1440-1819.2011.02236.x

[47] Achenbach TM , Rescorla LA. Manual for the ASEBA preschool forms & profiles. Burlington, VT, USA: ASEBA; 2003.

[48] Funabiki IY , Murai T . Standardization of a Japanese version of the Child Behavior Checklist for Ages 1½‐5 and the Caregiverteacher report form. Jpn J Child Adolesc Psychiatr. 2017;58:713–729.

[49] Package “mice” . 2022. Accessed June 8, 2024. Available from: https://cran.r-project.org/web/packages/mice/mice.pdf

[50] Newman L , Judd F , Olsson CA , Castle D , Bousman C , Sheehan P , et al. Early origins of mental disorder—risk factors in the perinatal and infant period. BMC Psychiatry. 2016;16:270. Published 2016 Jul 29 10.1186/s12888-016-0982-7 27473074 PMC4966730

[51] O'Donnell KJ , Bugge Jensen A , Freeman L , Khalife N , O'Connor TG , Glover V . Maternal prenatal anxiety and downregulation of placental 11β‐HSD2. Psychoneuroendocrinology. 2012;37:818–826. 22001010 10.1016/j.psyneuen.2011.09.014

[52] Glover V , O'Connor TG , O'Donnell K . Prenatal stress and the programming of the HPA axis. Neurosci Biobehav Rev. 2010;35:17–22. 19914282 10.1016/j.neubiorev.2009.11.008

