# Supplementary material for: Combination of taking neuropsychiatric medications and psychological distress in pregnant women, with behavioral problems in children at 2 years of age: The Tohoku Medical Megabank Project Birth and Three‐Generation Cohort Study
Source: PCN Rep. 2024 Jul 25;3(3):e226. doi: 10.1002/pcn5.226 (PMC11272827; doi:10.1002/pcn5.226)
Supplement: Supplementary file 1 — Supporting information. [file PCN5-3-e226-s001.docx]

| **Supplementary Table 1.** Number of mothers who took the neuropsychiatric medications during pregnancy | | |
| --- | --- | --- |
| Name of the medication | Number of mothers who took the medications during pregnancy | |
|  | From the onset of pregnancy to  early pregnancy | From early to mid-pregnancy |
| Alprazolam, n (%) | 10 (8.55) | 7 (9.09) |
| Amoxapine, n (%) | 3 (2.56) | 1 (1.30) |
| Bromazepam, n (%) | 4 (3.42) | 3 (3.90) |
| Clotiazepam, n (%) | 5 (4.27) | 5 (6.49) |
| Cloxazolam, n (%) | 1 (0.85) | 2 (2.60) |
| Diazepam, n (%) | 1 (0.85) | 4 (5.19) |
| Duloxetine Hydrochloride, n (%) | 3 (2.56) | 3 (3.90) |
| Ethyl Loflazepate, n (%) | 4 (3.41) | 3 (3.90) |
| Etizolam, n (%) | 9 (7.69) | 10 (12.99) |
| Escitalopram oxalate, n (%) | 5 (4.27) | 1 (1.30) |
| Fluvoxamine Maleate, n (%) | 4 (3.42) | 3 (3.90) |
| Hydroxyzine Hydrochloride, n (%) | 1 (0.85) | 1 (1.30) |
| Lorazepam, n (%) | 6 (5.13) | 3 (3.90) |
| Maprotiline Hydrochloride, n (%) | 2 (1.71) | 2 (2.60) |
| Mirtazapine, n (%) | 3 (2.56) | 2 (2.60) |
| Paroxetine Hydrochloride Hydrate, n (%) | 4 (3.42) | 2 (2.60) |
| Sertraline Hydrochloride, n (%) | 7 (5.98) | 4 (5.19) |
| Tofisopam, n (%) | 1 (0.85) | 1 (130) |
| Hangekobokuto, n (%) | 30 (25.64) | 9 (11.69) |
| Ryokeijutsukanto, n (%) | 1 (0.85) | 3 (3.90) |
| Kososan, n (%) | 5 (4.27) | 2 (2.60) |
| Saikokeishikankyoto, n (%) | 2 (1.71) | 1 (1.30) |
| Yokukansan, n (%) | 4 (3.42) | 4 (5.19) |
| Yokukansankachimpihange, n (%) | 2 (1.71) | 1 (1.30) |
| A total of 115 pregnant women who had taken neuropsychiatric medications from the onset of pregnancy to early pregnancy or early to mid-pregnancy were included in the table. Some pregnant women take multiple medications. | | |

| **Supplementary Table 2.** **Number of mothers who took neuropsychiatric medications during pregnancy according to psychological distress at mid-pregnancy.** | | |
| --- | --- | --- |
| Name of the medication | Number of mothers who took the medications during pregnancy | |
|  | K6<13 in mid-pregnancy | K6≥13 in mid-pregnancy |
| Alprazolam, n (%) | 16 (10.53) | 1 (2.38) |
| Amoxapine, n (%) | 4 (2.63) | 0 (0.0) |
| Bromazepam, n (%) | 7 (4.61) | 0 (0.0) |
| Clotiazepam, n (%) | 5 (3.29) | 5 (11.90) |
| Cloxazolam, n (%) | 0 (0.0) | 3 (7.14) |
| Diazepam, n (%) | 5 (3.29) | 0 (0.0) |
| Duloxetine Hydrochloride, n (%) | 3 (1.97) | 3 (7.14) |
| Ethyl Loflazepate, n (%) | 4 (2.63) | 3 (7.14) |
| Etizolam, n (%) | 15 (9.87) | 4 (9.52) |
| Escitalopram oxalate, n (%) | 6 (3.95) | 0 (0.0) |
| Fluvoxamine Maleate, n (%) | 5 (3.29) | 2 (4.76) |
| Hydroxyzine Hydrochloride, n (%) | 2 (1.32) | 0 (0.0) |
| Lorazepam, n (%) | 8 (5.26) | 2 (4.76) |
| Maprotiline Hydrochloride, n (%) | 0 (0.0) | 4 (9.52) |
| Mirtazapine, n (%) | 4 (2.63) | 1 (2.38) |
| Paroxetine Hydrochloride Hydrate, n (%) | 2 (1.32) | 4 (9.52) |
| Sertraline Hydrochloride, n (%) | 11 (7.24) | 0 (0.0) |
| Tofisopam, n (%) | 2 (1.32) | 0 (0.0) |
| Hangekobokuto, n (%) | 35 (23.03) | 5 (11.90) |
| Ryokeijutsukanto, n (%) | 4 (2.63) | 0 (0.0) |
| Kososan, n (%) | 7 (4.61) | 0 (0.0) |
| Saikokeishikankyoto, n (%) | 1 (0.66) | 2 (4.76) |
| Yokukansan, n (%) | 4 (2.63) | 3 (7.14) |
| Yokukansankachimpihange, n (%) | 2 (1.32) | 0 (0.0) |
| A total of 115 pregnant women who had taken neuropsychiatric medications from the onset of pregnancy to early pregnancy or early to mid-pregnancy were included in the table. Some pregnant women take multiple medications. | | |

| **Supplementary Table 3.** Comparison of characteristics of included and not included participants. | | | | |
| --- | --- | --- | --- | --- |
|  |  | Participants  included | Participants  not included | P-value* |
|  |  | n=10,296 | n = 11,454 |  |
| **Maternal age, n (%)** | <25 (year) | 548 (5.3) | 1,172 (10.2) | <0.001 |
|  | 25-30 (year) | 2,475 (24.1) | 3,079 (26.9) |  |
|  | 30-35 (year) | 3,946 (38.3) | 4,028 (35.2) |  |
|  | >35 (year) | 3,327 (32.3) | 3,175 (27.7) |  |
| **Parity, n (%)** | Nullpara | 4,876 (47.5) | 5,412 (47.4) | 0.886 |
|  | Multipara | 5,397 (52.5) | 6,016 (52.6) |  |
|  | Missing | 23 | 26 |  |
| **Maternal alcohol drink, n (%)** | Never | 4,655 (45.4) | 4,974 (46.2) | <0.001 |
|  | Fomer | 3,472 (33.9) | 3,814 (35.4) |  |
|  | Current | 2,119 (20.7) | 1,982 (18.4) |  |
|  | Missing | 50 | 684 |  |
| **Maternal smoking, n (%)** | Never | 6,554 (64.0) | 6,018 (55.9) | <0.001 |
|  | Stopped before pregnancy | 2,415 (23.6) | 2,503 (23.3) |  |
|  | Stopped after pregnancy | 1,108 (10.8) | 1,880 (17.5) |  |
|  | Current | 162 (1.6) | 358 (3.3) |  |
|  | Missing | 57 | 695 |  |
| **Educational attainment, n (%)** | High school graduate or less | 2,820 (30.6) | 1,594 (37.2) | <0.001 |
|  | Junior college or vocational college graduate | 3,570 (38.7) | 1,633 (38.1) |  |
|  | University graduate or above | 2,802 (30.4) | 1,049 (24.5) |  |
|  | Others | 24 (0.3) | 6 (0.2) |  |
|  | Missing | 1080 | 7172 |  |
| **Income, n (%)** | <4,000,000 (JPY/year) | 3,322 (33.6) | 3,857 (39.5) | <0.001 |
|  | 4,000,000-<5,999,999 (JPY/year) | 3,325 (33.6) | 3,069 (31.5) |  |
|  | ≥6,000,000 (JPY/year) | 3,238 (32.8) | 2,824 (29.0) |  |
|  | Missing | 411 | 1,704 |  |
| **Paternal smoking, n (%)** | Never | 3,102 (30.4) | 2,770 (25.9) | <0.001 |
|  | Stopped before pregnancy | 2,490 (24.4) | 2,129 (19.9) |  |
|  | Stopped after pregnancy | 274 (2.7) | 333 (3.1) |  |
|  | Current | 4,327 (42.5) | 5,463 (51.1) |  |
|  | Missing | 103 | 759 |  |
| **Child's gender, n (%)** | Male | 5,306 (51.5) | 5,981 (52.2) | 0.321 |
| JPY Japanese Yen  *Obtained using the chi-squared test. Chi-squared test was performed with missing participants excluded. For participants for whom each variable was missing, only the number of participants was reported. | | | | |

| **Supplementary Table 4.** The association of 4 categories of taking neuropsychiatric medications and maternal psychological distress (K6≥5) during pregnancy with children's behavioral problems at 2 years of age by multivariate logistic regression. | | | | | | | | | | | |
| --- | --- | --- | --- | --- | --- | --- | --- | --- | --- | --- | --- |
| Children’s behavioral problems  at aged 2 years | 4 categories of taking neuropsychiatric medications and maternal psychological distress during pregnancy | | | | | | | | | | |
|  | None  (n = 7,390) | |  | Medications only  (n = 49) | |  | K6≥5 only  (n = 2,791) | |  | Both  (n = 66) | |
|  | Crude  OR (95% CI) | Adjusted  OR  (95% CI)^a^ |  | Crude  OR (95% CI) | Adjusted  OR  (95% CI)^a^ |  | Crude  OR (95% CI) | Adjusted  OR  (95% CI)^a^ |  | Crude  OR (95% CI) | Adjusted  OR  (95% CI)^a^ |
| Externalizing problems | Ref | |  | 1.55  (0.59-3.37) | 1.52  (0.64-3.61) |  | 2.58  (2.28-2.93) | 2.36  (2.08-2.68) |  | 2.46  (1.25-4.46) | 2.07  (1.09-3.93) |
| Internalizing problems | Ref | |  | 1.00  (0.24-2.75) | 0.94  (0.30-3.07) |  | 2.43  (2.11-2.81) | 2.19  (1.89-2.54) |  | 2.74  (1.31-5.17) | 2.22  (1.11-4.42) |
| ^a^Adjusted for maternal age at delivery, parity, educational attainment, household income, maternal alcohol intake, maternal cigarette smoking, paternal cigarette smoking, child's sex | | | | | | | | | | | |
| K6 *Kessler Psychological Distress Scale*, 95% CI *95% confidence interval*, OR *Odds ratio* | | | | | | | | | | | |

| **Supplementary Table 5.** The association of 4 categories of taking neuropsychiatric medications and maternal psychological distress (K6≥9) during pregnancy with children's behavioral problems at 2 years of age by multivariate logistic regression. | | | | | | | | | | | |
| --- | --- | --- | --- | --- | --- | --- | --- | --- | --- | --- | --- |
| Children’s behavioral problems at aged 2 years | 4 categories of taking neuropsychiatric medications and maternal psychological distress during pregnancy | | | | | | | | | | |
|  | None  (n = 9,219) | |  | Medications only  (n = 79) | |  | K6≥9 only  (n = 962) | |  | Both  (n = 36) | |
|  | Crude  OR (95% CI) | Adjusted  OR  (95% CI)^a^ |  | Crude  OR (95% CI) | Adjusted  OR  (95% CI)^a^ |  | Crude  OR (95% CI) | Adjusted  OR  (95% CI)^a^ |  | Crude  OR (95% CI) | Adjusted  OR  (95% CI)^a^ |
| Externalizing problems | Ref | |  | 1.48  (0.74-2.69) | 1.40  (0.73-2.67) |  | 2.87  (2.43-3.37) | 2.52  (2.14-2.98) |  | 2.20  (0.89-4.77) | 1.76  (0.76-4.08) |
| Internalizing problems | Ref | |  | 1.43  (0.63-2.81) | 1.25  (0.60-2.64) |  | 2.55  (2.11-3.07) | 2.27  (1.87-2.75) |  | 2.05  (0.70-4.84) | 1.72  (0.66-4.52) |
| ^a^Adjusted for maternal age at delivery, parity, educational attainment, household income, maternal alcohol intake, maternal cigarette smoking, paternal cigarette smoking, child's sex | | | | | | | | | | | |
| K6 *Kessler Psychological Distress Scale*, 95% CI *95% confidence interval*, OR *Odds ratio* | | | | | | | | | | | |
